# Can repeat IVF/ICSI cycles compensate for the natural decline in fertility with age? an estimate of cumulative live birth rates over multiple IVF/ICSI cycles in Chinese advanced-aged population

**DOI:** 10.18632/aging.203055

**Published:** 2021-05-20

**Authors:** Fang Gu, Simin Ruan, Chenxiang Luo, Ying Huang, Lu Luo, Yanwen Xu, Canquan Zhou

**Affiliations:** 1 Department of Obstetrics and Gynecology, Reproductive Medical Center, The First Affiliated Hospital of Sun Yat-Sen University, Guangzhou 510080, China; 2 Department of Medical Ultrasonics, Institute of Diagnostic and Interventional Ultrasound, The First Affiliated Hospital of Sun Yat-Sen University, Guangzhou 510080, China; 3 Department of Obstetrics and Gynecology, The First Affiliated Hospital of Sun Yat-Sen University, Guangzhou 510080, China; 4 Key Laboratory of Reproductive Medicine of Guangdong Province, Guangzhou 510080, China

**Keywords:** advanced maternal age, POSEIDON criteria, ovarian reserve, cumulative live birth rate, poor ovarian response

## Abstract

In order to find out to what extent ovarian aging could be compensated by the *in vitro* fertilization/intracytoplasmic sperm injection (IVF/ICSI) treatments, a total of 4102 women above the age of 35 undergoing 6489 complete cycles from 2009 to 2015 with follow-up visits until 2017 were retrospectively analyzed. Cumulative live birth rates (CLBRs) across multiple IVF/ICSI cycles were compared in the study population stratified by age and ovarian reserve (classified by the POSEIDON criteria). Younger patients (aged between 35 and 40) could well benefit from repeat IVF treatments, with the optimal CLBRs ranging from 62%-72% for up to four complete cycles. However, the CLBRs sharply declined to 7.7%-40% in older patients (>40yrs). In light of ovarian reserve, the optimal-estimated-four-cycle CLBR of younger patients (35-40yrs) in POSEIDON group 2 could approached to those with normal ovarian response (non-POSEIDON), with 57.3%-70% versus 74.5%-81% respectively. However, the CLBR of older patients (>40yrs) in POSEIDON group 2 only reached 50% of their counterparts. Extending the number of IVF cycles beyond three or four is effective for advanced-aged women, especially in younger normal responders (non-POSEIDON) and unexpected poor/suboptimal responders (POSEIDON group 2). The real turning point at which female fecundity dropped after multiple IVF cycles is at the age of 40.

## INTRODUCTION

Since China officially abolished its long held one-child policy in 2016, 90 million Chinese couples are eligible to have a second child. [1]. However, among these prospective second-child mothers, 60% of them are advanced maternal age (AMA) women who are above the age of 35, and more than half are 40 years and older [1, 2]. This is also the case globally, as women over 35 years old undergoing *in vitro* fertilization/intracytoplasmic sperm injection (IVF/ICSI) treatments now represent the most rapidly growing population [3–5]. However, whether the age-related decline in fertility can be compensated by IVF/ICSI is still controversial as age is the most determinant of oocyte quantity and quality [6, 7]. Since IVF/ICSI is an expensive and time-consuming treatment procedure without being covered by the health insurance in many countries, the chance of success should be carefully discussed with AMA patients before treatment initiation. Therefore, to help clinicians to provide accurate, individualized counselling and to help patients to build realistic expectations for their reproductive outcomes, data are needed to estimate the efficacy of IVF in AMA women.

The cumulative live birth rate (CLBR) over multiple complete IVF cycles calculates the chance of a live birth over several consecutive ovarian stimulations, including all subsequent fresh and frozen embryo transfers. It is especially pertinent to an AMA couples’ who might need more than one complete IVF/ICSI cycle before having a live birth. [8–10]. Population-based studies have shown that female age plays a major role in the CLBR within one or over multiple stimulation cycles [11–14]. However, the age point at which the CLBR sharply drops and defines “how old is too old” for IVF/ICSI treatments is still up for debate. In addition, ovarian reserve is another essential variable which has rarely been calculated in prediction models [15–17]. A few studies have assessed the CLBR in relation to the number of oocytes retrieved within different age groups [18–20]. However, the number of oocytes in a single stimulation cycle could not fully reflect the ovarian reserve as it is prone to being affected by the ovarian stimulation (OS) protocol used [20–22]. Therefore, in this study we included the POSEIDON (Patient-Oriented Strategies Encompassing IndividualizeD Oocyte Number) criteria, a novel classification model which combines age, ovarian reserve markers and the response to a previous OS to predict the prognosis for AMA patients who undergo IVF treatment [23, 24].

Thus, this study evaluated estimated CLBRs over multiple complete IVF/ICSI cycles in Chinese AMA women stratified into different age groups. Moreover, to further create homogeneous populations, we applied the POSEIDON criteria to define ovarian reserve of the studied population.

## RESULTS

### Characteristics of the study population

**Figure 1:**
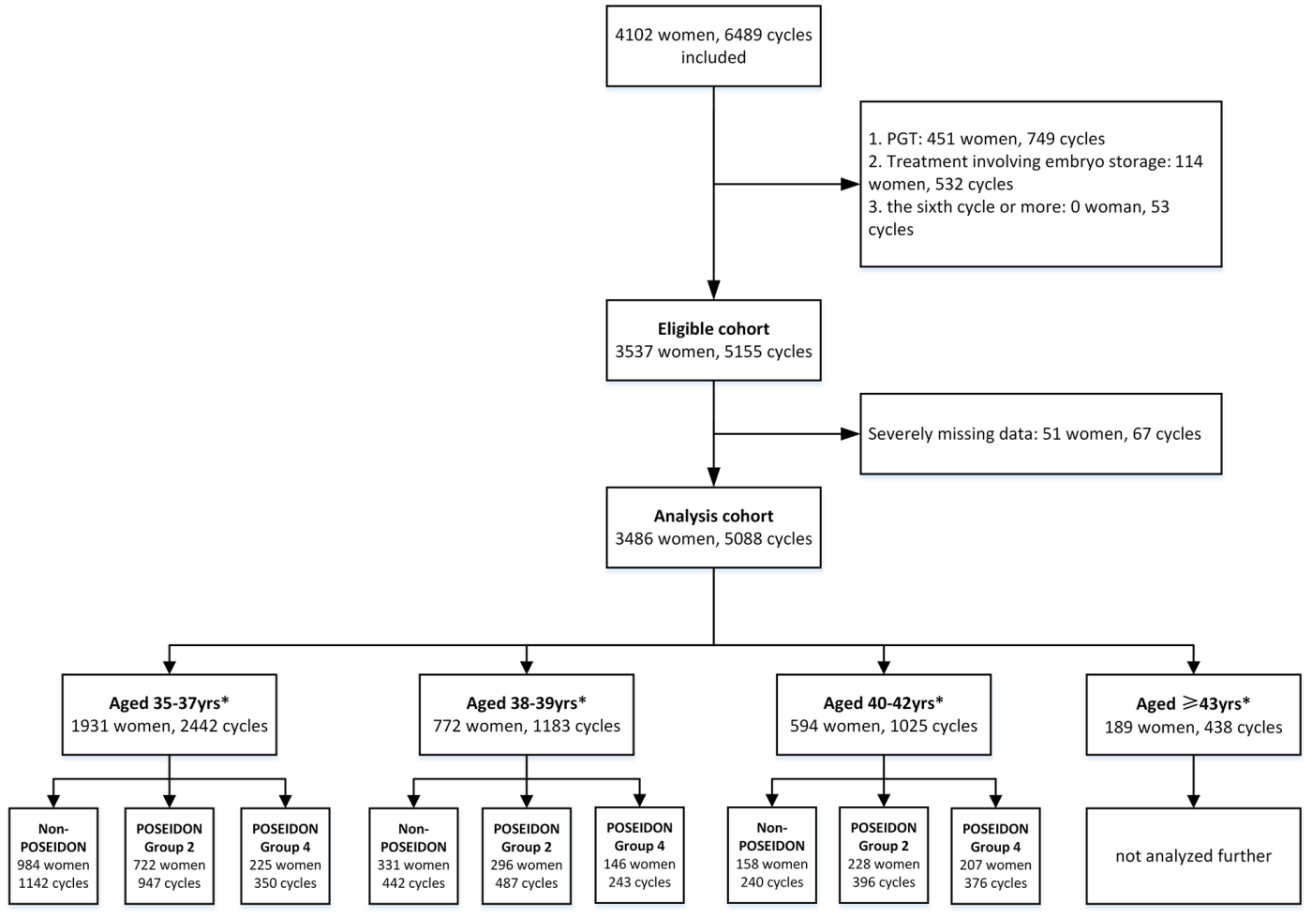
**Flowchart and data processing of the study population.** Notes: *data of the fifth cycle were not included for subgroup analysis because of few cases. PGT, preimplantation genetic testing; ART, assisted reproductive technology.

A total of 4102 Chinese AMA women, undergoing 6489 IVF cycles were included in this analysis. Of these, 616 patients were excluded for the following reasons: preimplantation genetic testing (PGT) cycles, embryo storage, missing data, etc. Finally, the study population consisted of 3,486 Chinese AMA women undergoing a total of 5088 IVF/ICSI cycles, including 1931 women aged 35-37 years, 772 women aged 38-39 years, 594 women aged 40-42 years and 189 women aged ≥43 years. Patients in each age strata were subsequently categorized into the following subgroups: POSEIDON group 2, POSEIDON group 4, and non-POSEIDON group. A flow chart with details of the cohort analyzed is presented in Figure 1.

Baseline and treatment characteristics are shown in Table 1. Among the 3486 women, the mean (±SD) body mass index (BMI) was 22.0±2.7 kg/m^2^, while 79.5% had normal BMI. The median (IQR) of infertility duration was 5.0 (3.0-9.0) years. The mean gravidity was 1.3±1.4 and 63.6% of the patients were secondary infertility. The most common infertility factor was pelvic and tubal factor (48.9%). Ovarian reserve declined as age increased, the rate of low prognosis (POSEIDON group 2 and group 4) increased from 49% in women aged 35-37yrs to around 90% in women above 43 yrs. Gonadotrophin-releasing hormone (GnRH) long agonist protocol was the most common ovarian stimulation protocol (67.5%), followed by GnRH antagonist protocol (12.3%) and GnRH short agonist protocol (12.3%). Mild stimulation protocol was more frequently used among women aged over 43 (19.1%). A total of 3409 (67.0%) underwent IVF, while the remaining 1678 (33.0%) was ICSI cycle. The mean number of oocytes retrieved and embryos available for transfer in the first stimulation cycle were 8.8±6.8 and 3.6±3.0, both of which decreased as age increased, from 10.5±7.2 oocytes and 4.5±3.4 embryos in the group of 35-37 years old to 4.4±4.3 oocytes and 2.1±1.8 embryos in the group above 43 years old. No more than three embryos were transferred to each patient at a time.

**Table 1:** Characteristics of study population.

**Characteristics**	**35-37yrs**	**38-39yrs**	**40-42yrs**	**≥43yrs**	**All AMA patients**
No. of women	1931	772	594	189	3486
No. of cycles	2442	1183	1025	438	5088
BMI (kg/m^2^)	21.7±2.7	22.2±2.7	22.2±2.7	22.5±2.8	22.0±2.7
<18.5	173(9.0)	47(6.1)	32(5.4)	10(5.3)	262(7.6)
18.5-24.9	1528(79.7)	602(78.4)	479(81.2)	146(77.2)	2755(79.5)
≥25	217(11.3)	119(15.5)	79(13.4)	33(17.5)	448(12.9)
Duration of infertility (yrs.)	5.0 (3.0-8.0)	6.0 (3.0-10.0)	5.0 (2.0-10.0)	4.0 (2.0-9.5)	5.0 (3.0-9.0)
Parity≥1(%)	397 (20.6)	220 (28.5)	225 (37.9)	102 (54.0)	944 (27.1)
bFSH(IU/L)	6.3±2.6	6.5±3.0	7.4±3.7	7.9±4.2	6.6±3.0
≤10	1808 (93.6)	711 (92.1)	505 (85.0)	145 (76.7)	3169(90.9)
>10	123(6.4)	61 (7.9)	89 (15.0)	44 (23.3)	317 (9.1)
bFSH/bLH	2.2±1.1	2.5±1.6	2.6±1.2	2.7±1.9	2.4±1.3
bE2(pmol/L)	144.2±113.0	143.9±88.1	154.5±114.9	192.3±269.4	148.3±122.6
Type of infertility (%)					
Primary	780(41.4)	258 (33.4)	169 (28.5)	43 (22.8)	1270(36.4)
Secondary	1131(58.6)	514 (66.6)	425 (71.5)	146 (77.2)	2216(63.6)
Cause of infertility (%)					
Unexplained	17 (0.9)	6 (0.8)	5 (0.9)	2 (1.1)	30 (0.9)
Severe endometriosis	84 (4.4)	22 (2.8)	24 (4.0)	3 (1.6)	133 (3.8)
Pelvic and tubal factor	949 (49.1)	378 (49.0)	292 (49.2)	88 (46.6)	1707(48.9)
Ovulatory dysfunction	24 (1.2)	8 (1.0)	9 (1.5)	7 (3.7)	48 (1.4)
Male factor	461 (23.9)	179 (23.2)	127 (21.4)	42 (22.2)	809 (23.2)
combined	396 (20.5)	179 (23.2)	137 (23.1)	47 (24.9)	759 (21.8)
Ovarian reserve					
Non- POSEIDON	984(51.0)	330(42.7)	159(26.8)	20(10.6)	1493(42.8)
POSEIDON group 2	722(37.4)	296(38.3)	228(38.4)	75(39.7)	1321(37.9)
POSEIDON group 4	225(11.7)	146(18.9)	207(34.8)	94(49.7)	672(19.3)
Ovarian stimulation protocol					
GnRH long agonist	1972(80.8)	812(68.6)	522(51.0)	126(28.6)	3432(67.5)
GnRH antagonist Minimal stimulation	178(7.3)	143(12.1)	185(18.1)	120(27.3)	626(12.3)
	72(2.9)	52(4.4)	84(8.2)	84(19.1)	292(5.7)
GnRH short agonist	171(7.0)	152(12.8)	205(20.0)	99(22.5)	627(12.3)
others	49(2.0)	24(2.0)	27(2.6)	11(2.5)	111(2.2)
Treatment protocol (%)					
IVF	1648(67.5)	790 (66.8)	695 (68.0)	276(62.7)	3409(67.0)
ICSI	794(32.5)	393 (33.2)	327 (32.0)	164 (37.3)	1678(33.0)
Number of oocytes in the 1^st^ OPU cycle	11.1±7.4	9.5±6.5	7.0±5.6	4.6±4.3	9.7±7.0
Average number of retrieved oocytes	10.5±7.2	9.0±6.3	6.5±5.2	4.4±4.3	8.8±6.8
Number of available embryos in the 1^st^ OPU cycle	4.5±3.4	3.8±2.9	3.0±2.4	2.1±1.8	4.0±3.2
Average number of available embryos	4.3±3.3	3.6±2.7	2.8±2.3	2.0±1.8	3.6±3.0

Data are presented as mean ± SD, median (IQR) and n (%). AMA, advanced maternal age; BMI, body mass index; bFSH, basal follicle stimulating hormone; bLH, basal luteinizing hormone; bE2, basal estradiol; IVF, *in-vitro* fertilization; ICSI, intracytoplasmic sperm injection; OPU, Ovum Pick-up.

### CLBRs in different age groups

A total of 1414 women had live births after IVF/ICSI during the study period, accounting for a total LBR of 40.6%. The LBR within the first cycle was 32.2% (95% confidence interval [CI] 30.6%-33.7%), which gradually decreased to the fifth cycle with 2.6% (95%CI 2.5%-7.7%) per cycle. However, the CLBRs increased as the number of cycles increased. The optimal CLBR estimate after five cycles was 58.0%, the age-adjusted estimated CLBRs were 49.1% and the conservative-estimated CLBRs were 41.7%. The estimated CLBRs increased up to the 3^rd^ IVF/ICSI cycles and then reached a plateau [19] (increase of ≤5%) after the 4^th^ cycle (Supplementary Table 1).

**Figure 2:**
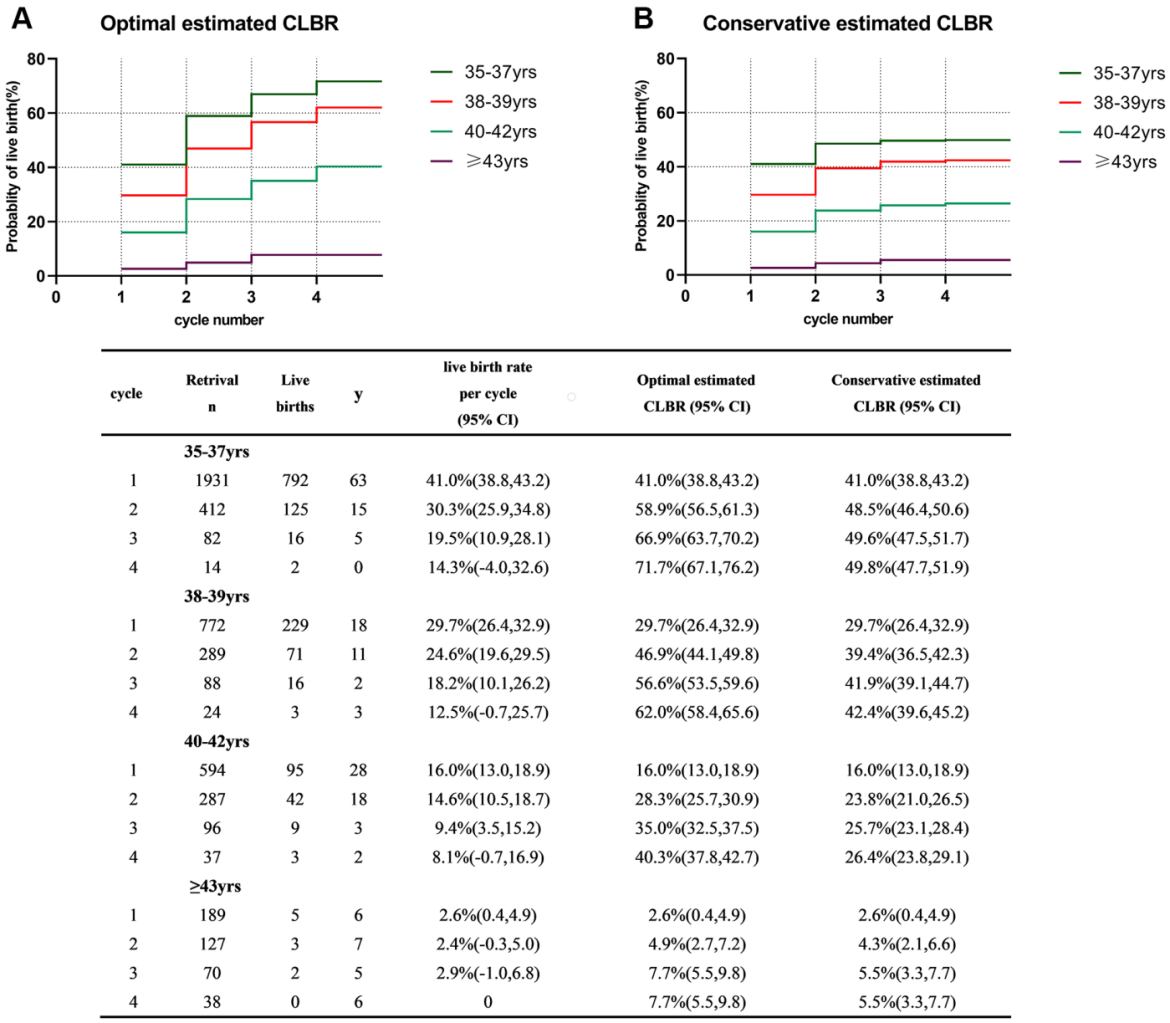
**Cumulative live birth rates in different age groups.** (**A**) The optimal estimated cumulative live birth rates over 4 complete cycles. (**B**) The conservative estimated cumulative live birth rates over 4 complete cycles. CLBR, cumulative live birth rate. Notes: *The optimal estimated CLBR assumes that women who discontinued IVF/ICSI treatments would have live-birth rate similar to those continuing treatments. The conservative estimated CLBR assumes that women who discontinued IVF treatments would have a live-birth rate of zero if they continued treatments.

Figure 2 shows the optimal and conservative estimated CLBRs for each age-group. Both the optimal and conservative CLBRs significantly decreased with increasing age (*P*<0.001). Women in the group of 35-37 years had the best prognosis, with a CLBR of 41% after the first cycle and the optimal and conservative estimated CLBRs continuing to increase up to the 4^th^ cycle to 71.1% and 49.8%, respectively. For women in the group of 38-39 years, although the CLBR declined significantly in the first cycle compared with that of the group of 35-37 years, they benefitted from repeat stimulation cycles. Their probability of success could reach acceptable figures between 62.0% and 42.4%. However, the LBR in the group of 40-42 years sharply declined to only 16.0% in the first stimulation cycle and the maximal likelihood of live birth was 40.3% after four stimulation cycles. For women older than 43 years old, the LBR per cycle was extremely low as optimal and conservative estimated CLBRs after the 4^th^ cycle was only 7.7% and 5.5% respectively.

### CLBRs according to ovarian reserve in different age groups

**Figure 3:**
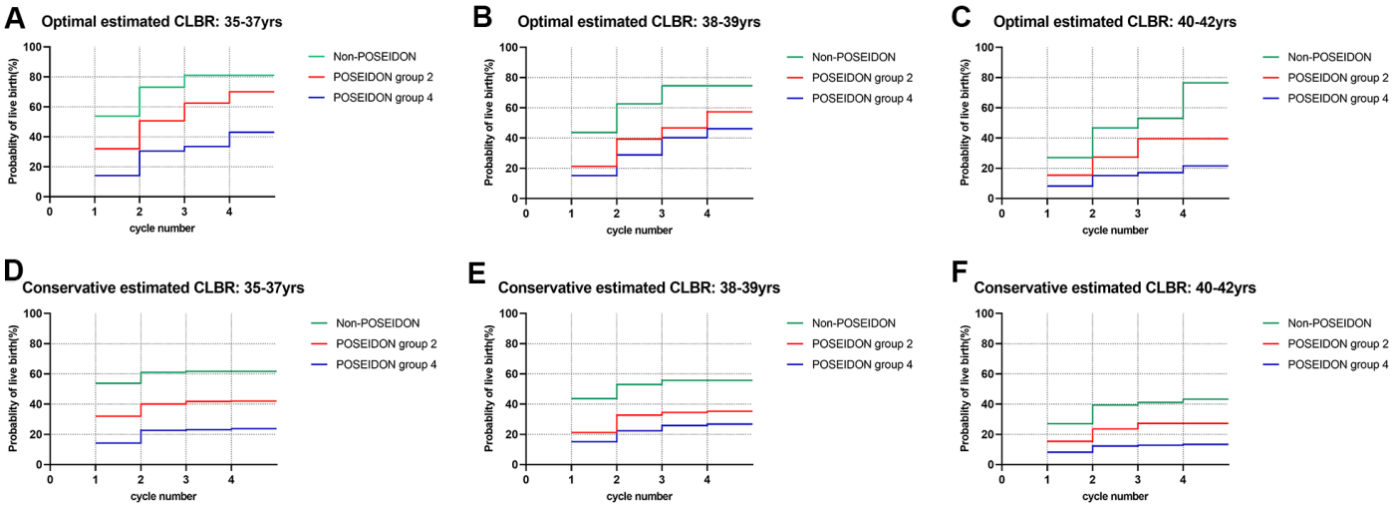
**Cumulative live birth rates according to ovarian reserve in different age groups.** (**A**–**C**) The optimal estimated cumulative live birth rates stratified by ovarian reserve in different age groups. (**D**–**F**) The conservative estimated cumulative live birth rates stratified by ovarian reserve in different age groups. CLBR, cumulative live birth rates. Notes: *The optimal estimated CLBR assumes that women who discontinued IVF/ICSI treatments would have live-birth rate similar to those continuing treatments. The conservative estimated CLBR assumes that women who discontinued IVF treatments would have a live-birth rate of zero if they continued treatments.

Estimated CLBRs with different ovarian reserve for a given age group are graphically depicted in Figure 3. As shown in Figure 3, in all age groups, the LBR of the first cycle as well as the CLBRs over multiple cycles in the non-POSEIDON group were significantly higher than in POSEIDON group 2 (unexpected poor/suboptimal responder), followed by POSEIDON group 4 (expected poor responder) (*P<*0.001). However, women aged 35-37 yrs. and 38-39 yrs. of POSEIDON group 2 had a four-cycle CLBR of ~70% and ~57.3% optimally, which came close to those non-POSEIDON patients having CLBRs of ~81% and ~74.5% (Supplementary Tables 2–4). In contrast, patients aged 40-42 yrs. had a four-cycle CLBR of ~39.5%, which was 50% lower than the non-POSEIDON group.

LBRs for patients in POSEIDON group 4 were much lower than their counterparts in each age strata and the highest LBR per IVF cycle was 15.1%. However, some benefit from increasing the number of cycles could still be seen. Thus, the CLBR rose from 14.2%-15.1% for the first cycle to 43%-46.1% after the 4^th^ cycle in patients below 40 years of age. In contrast, the prognosis for older patients (≥40yrs) of POSEIDON group 4 was poor and their maximal estimated CLBR was 21.5%, only, even after four complete IVF cycles.

### Univariate and multivariate COX regression analysis

Table 2 presented crude hazard ratios (HRs) and adjusted HRs for associations between clinical parameters and CLBR. In univariate analysis, age, ovarian reserve, parity, type of infertility and infertility factor were significantly associated with CLBR (*P* < 0.05). As expected, multivariate analysis adjusting for all potential confounders showed that both female age [38-39yrs.: aHR=0.67 (95%CI 0.59-0.77, *P* < 0.001); 40-42yrs.: aHR=0.40 (95%CI: 0.33-0.48, *P* < 0.001); ≥43yrs.: aHR=0.08(95%CI: 0.05-0.14, *P* < 0.001; all vs. 35-37yrs.] and ovarian reserve [POSEIDON group 2: aHR=0.51, 95%CI: 0.46-0.58, *P* < 0.001; POSEIDON group 4: aHR=0.26, 95%CI: 0.21-0.31, *P* < 0.001; both vs. POSEIDON group 4] were independent predictors of CLBR. Male factor infertility was positively associated with CLBR and this effect remained statistically significant in multivariate analysis (aHR=1.20, 95%CI: 1.02-1.42, *P* = 0.025).

**Table 2:** Univariate and multivariate Cox regression analysis of prognostic factors for live-birth in advanced age women.

**Risk factors**	**Unadjusted HR** **(95%CI)**	***P***	Adjusted HR^*^ (95%CI)	***P***
**Age**				
35-37yr	Reference		Reference	
38-39yr	0.63(0.55, 0.71)	<0.001	0.67(0.59, 0.77)	<0.001
40-42yr	0.31(0.26, 0.38)	<0.001	0.40(0.33, 0.48)	<0.001
≥43yr	0.05(0.03 0.09)	<0.001	0.08(0.05, 0.14)	<0.001
**Ovarian reserve**				
Non-POSEIDON	Reference		Reference	
POSEIDON group 2	0.46(0.41, 0.51)	<0.001	0.51(0.46, 0.58)	<0.001
POSEIDON group 4	0.18(0.15, 0.22)	<0.001	0.26(0.21, 0.31)	<0.001
**BMI(kg/m^2^)**				
<18.5	0.97(0.79, 1.20)	0.780	0.93(0.75, 1.15)	0.487
≥25	1.05(0.90,1.23)	0.512	1.14(0.97, 1.33)	0.109
18.5-24.9	Reference		Reference	
**Parity≥1**	0.82(0.73, 0.93)	0.002	1.02(0.89, 1.17)	0.773
**Type of infertility** (primary vs. secondary)	0.89(0.80, 0.99)	0.030	1.01(0.89, 1.14)	0.923
**Treatment protocol** (IVF vs. ICSI)	0.97(0.87, 1.08)	0.552	0.93(0.81, 1.07)	0.293
**Cause of infertility**				
Unexplained	1.26(0.67, 2.37)	0.474	1.19(0.63, 2.23)	0.597
Severe endometriosis	1.05(0.78, 1.42)	0.752	1.01(0.74, 1.36)	0.992
Pelvic and tubal factor	1.13(0.99, 1.30)	0.075	1.05(0.90, 1.21)	0.557
Ovulatory dysfunction	0.94(0.57, 1.55)	0.797	1.01(0.61, 1.68)	0.966
Male factor	1.30(1.11, 1.51)	0.001	1.20(1.02,1.42)	0.025
Combined	Reference		Reference	

*adjusted for female age, ovarian reserve, female BMI, parity, type of infertility, cause of infertility and treatment protocol.

HR=hazard ratio; CI=confidence interval; BMI=body mass index.

## DISCUSSION

### Main findings

This study explored the CLBR in 5088 IVF/ICSI cycles performed in AMA patients. The results show that CLBRs increased significantly by extending the number of IVF cycles, and that the increase was most evident during the first three cycles, followed by a plateau (increase<5%). Subgroup analyses showed that CLBRs declined with increasing age and decreasing ovarian reserve. However, the CLBRs of POSEIDON group 2 could more or less “catch up” with the non-POSEIDON group after multiple IVF/ICSI cycles for patients below 40 years of age. Findings from the current study support the benefit of extending the number of IVF cycles up to three or four in AMA women below 43, especially in younger (<40yrs) normal responders (non-POSEIDON) and unexpected poor/suboptimal responders (POSEIDON group 2).

It has been seen that LBRs per cycle and the CLBRs over multiple cycles decrease sharply with the increase in the age of a woman predominantly above 35 years of age [11–14]. Our results are in agreement with these studies and multivariate COX regression analysis confirmed that age was an independent factor negatively associated with CLBR. With an increase by 2-3 years of age, the CLBR presented a significant decline. The distinct impact of female age on the reproductive outcome of an ART cycle is determined not only by the age-related decrease in oocyte quantity [25], but also by the accelerated decline in oocyte quality beginning around the age of 35 and being further intensified above the age of 40. This is well explained by the rising chances of aneuploid embryos from 34.5% at the age of 35 to 58.2% at the age of 40, and increases dramatically to 83.4% at the age of 43 [26], leading to a corresponding decrease in implantation rate [27] and pregnancy rate [28], as well as an increase in miscarriage rates [28, 29].

However, an ongoing debate is still the timing of the “advanced reproductive age”, at which the likelihood of conception greatly decreases even after IVF treatment. Current opinions suggest women >35 years of age to be AMA because the number of euploid embryos decreases sharply, leading to low implantation rates, higher miscarriage rates and poorer perinatal outcomes [30]. However, the results of the present study suggest that the prognosis of AMA women below the age of 40 is good after repeated IVF treatment. In contrast, the benefit of repeated stimulation sharply declines for patients above the age of 40. The optimal-four cycle estimated CLBR significantly dropped by 35% from the age of 38-39 to 40-42 years, and then progressively declined by 81% at the age of 43 compared to the age of 40-42 years counterparts. Hence, our data suggest that the real turning point at which the chance of live birth significantly dropped after multiple IVF cycles is at the age of 40. Although consistent with the Bologna criteria [31], our cut-off point for “advanced reproductive age” is higher as suggested by others - 35 [7] or 38 years [20]. This may be attributed to a high proportion of multipara in our study who were assumed to have a less steep decline in fecundability [32].

Women above the age of 43 had an extremely low LBR of only 2.6% after their first complete cycle. The CLBR plateaued at 7% maximally even after up to four cycles, which is in line with previous studies reporting that women above 43 years of age do not benefit in terms of CLBR irrespective of ovarian reserve and multiple IVF cycles [20]. As no effective interventions exist to counteract the age-related reduced oocyte quality and quantity, patients above 43 years of age should have detailed consultation regarding the low likelihood of reproductive success before starting an IVF treatment.

Ovarian reserve is another significant factor that is independently associated with the live birth rate. Our study combined the number of oocytes retrieved in the previous cycle and antral follicle count (AFC) to define ovarian reserve and stratify patients using the POSEIDON criteria, which is a recently proposed new stratification for low-prognosis patients in IVF/ICSI treatment, aiming to define more homogenous populations to predict prognosis and guide patient-tailored therapeutic approach. Consistent with the current literature [33–35], the CLBR was highest in non low-prognosis (non-POSEIDON) women, followed by POSEIDON Group 2 and POSEIDON Group 4. This result was robust even after adjusting for multiple confounders, including female age. Our study constructed a predictive model of CLBR of AMA patients in relation of different age strata (stratified by 2-year intervals) and ovarian reserve and we were delighted to find out that women with normal or unexpected poor response (non-POSEIDON and POSEIDON Group 2) could benefit more than those with expected low prognosis (POSEIDON Group 4) from extending the number of IVF cycles. Although both statistically significantly different, in the younger (<40years) POSEIDON group 2, their CLBR came close to those of the non-POSEIDON group after four complete cycles. Yet the optimal-estimated-four cycle CLBR of older POSEIDON group 2 patients (≥ 40 years) only reached half of their non-POSEIDON counterparts. This implied that the age-related decline in oocyte quality had a much more significant impact on the CLBR than oocyte quantity [33].

### Strengths and limitations

The current study has linked fresh and frozen embryo transfers to obtain estimates of CLBR across repeated stimulation cycles in advanced reproductive-aged women. It provides AMA women an age-stratified revised prognosis for chances of live birth based on the ovarian reserve. Although numerous studies evaluated CLBR around the world, most were conducted before 2014 [11–14, 16, 18–20]. Therefore, our results provide updated predictive figures for decision making in AMA women. The estimated CLBRs of the present analysis are similar to those reported by DEVESA [20] but higher when comparing to other reports [12–14].

Another strength of the study is that we included patients’ detailed information such as BMI, gravidity and parity which many population-based studies did not [11–14]. As it has been proven that BMI and prior live birth history are closely related to CLBR [32, 36], our study also provides new data focusing on the Asian race with generally lower BMI and higher gravidity and parity.

A weakness of the study is the high discontinuation rates after each cycle, a common problem while looking at cumulative outcomes. Reasons for discontinuation are mainly the high cost and emotional stress of repeat IVF treatments, especially in AMA women who are supposed to have a poorer prognosis. Thus, it has been assumed that for each 5-year increase in female age, the odds of not pursuing treatment increases by 77% [37]. Therefore, as seen in our data, as well as in previous studies, the range between optimal and conservative estimates is remarkable [11, 13]. Another weakness is the fact that we did not take Anti-Mullerian hormone (AMH) into account while evaluating the ovarian reserve because AMH was not used as a regular ovarian reserve test for patients during the study period. Instead, AFC, another reliable and accurate marker that has been proven to exhibit equivalent performance characteristics in predicting ovarian response as AMH was used [38].

In conclusion, the current study provides the first set of relevant data on CLBRs over multiple IVF cycles in AMA women categorized by age and ovarian reserve. Our findings support the efficacy of extending the number of cycles up to three or four until the age of 43 and recommendations should be given individually considering the age and ovarian reserve. Women above the age of 43 is not cost-effective to continue repeated IVF treatment using their own oocytes. Further work is required to move towards tailored protocols to maximize the IVF success rate of each age-specific POSEIDON group without compromising safety.

## MATERIALS AND METHODS

### Study participants and grouping

This was a retrospective single-center cohort study. Women above the age of 35 who initiated IVF treatment at Reproductive Center of the First Affiliated Hospital of Sun Yet-sen University in China between 1st January 2009 and 30th November 2015 were included in the study, and live birth outcome data were collected up to May 2017. We excluded PGT cycles and cycles initiated for embryo banking or fertility preservation. Cycles with missing data deletion were also excluded. Ethical approval for this study was obtained from the Institutional Review Board of the First Affiliated Hospital, Sun Yat-sen University (No.2016208).

**Figure 4:**
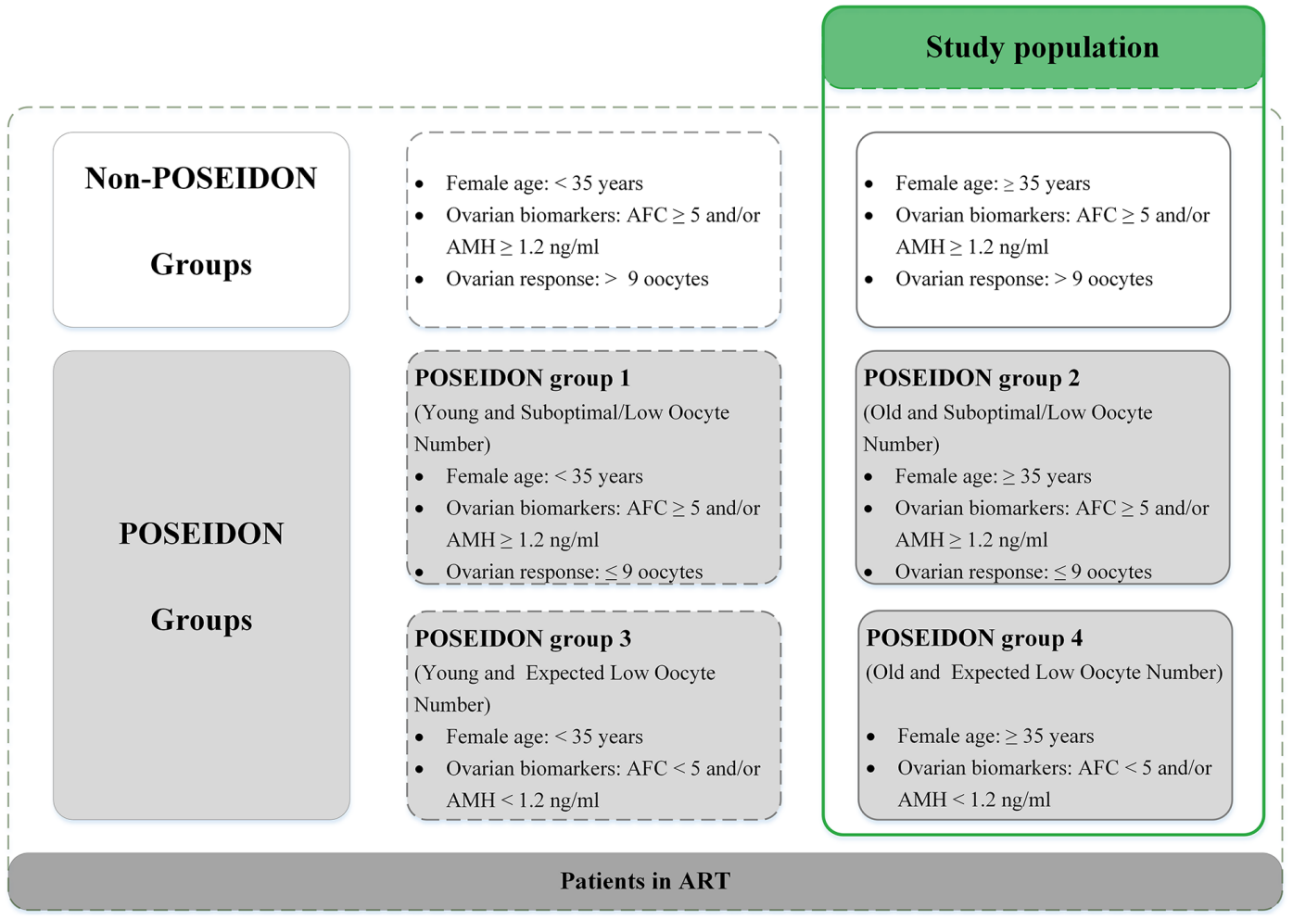
**Patient-oriented strategies encompassing individualized oocyte number (POSEIDON) criteria of low prognosis patients in assisted reproductive technology (ART).** Notes: Patients in the green table are the study population. AFC, antral follicle count; AMH, anti-Müllerian hormone.

To investigate how age influenced CLBRs, patients were categorized into four age groups [11]: 35-37 years; 38-39 years; 40-42 years and ≥43 years. To further explore the impact of the ovarian reserve on IVF/ICSI outcomes, we performed a subgroup analysis stratified by AFC and the number of oocytes retrieved during the first IVF cycle according to the POSEIDON criteria [23] in each age group. POSEIDON group 2 was defined as age≥35, AFC≥5, and previous number of oocytes retrieved<9. POSEIDON group 4 was defined as age≥35 and AFC<5 [23]. Women in whom ≥9 oocytes were retrieved during their first cycle [39] were defined as non-POSEIDON patients (Figure 4).

### Outcome measures

Live birth was defined as an infant born showing any sign of life after 28 weeks gestation [40]. Deliveries of multiple pregnancies were counted as one live birth. LBR and CLBR were defined as the probability of at least one live birth resulting from one and multiple stimulation cycles encompassing the fresh and subsequent frozen embryo transfers, respectively. As very few patients (n=38) underwent a fifth cycle and the data were too sparse to provide robust estimates, we only calculated the CLBR up to the fourth cycle in the subsequent analysis. The primary outcome was the CLBRs up to 1-4 IVF cycles in each age group, answering the question of the chance of a live birth with repeat IVF cycles.

The secondary outcome was the CLBRs in the same age group but with different ovarian reserve, which answered the question that to what extent ovarian reserve could play a part in the probability of live birth.

### Statistical analysis

We carried out three methods to estimate the CLBR in order to deal with the censor data for patients who did not return for treatment. The “Optimal” estimate assumes that women who do not return for subsequent IVF treatment have the same chance of a live birth as those who return for treatment. In contrast, the “conservative” estimate assumes that women who do not to return for further treatment will have a live birth rate of zero. To obtain a more realistic value, we calculated an age-adjusted estimate which was between these two extremes. According to Smith’s computing method [11], we assumed that 47% of women who discontinued IVF treatment were attributed to an age-related poor prognosis and subsequently that their LBRs would have been zero, had they continued, whereas the other 53% would have similar LBRs with those who continued treatment. Within our dataset, some patients eventually did continue treatment, but their next stimulation cycle occurred beyond the study period (after December 2015). We excluded these cycles (described as “y” in all the tables) and made adjustments for censoring to reduce the bias as Smith et al. reported [11].

For each group, descriptive data were expressed as mean ± standard deviation (SD), median (interquartile range, IQR) or number (percentage). Analysis of variance (ANOVA) or Kruskal-Wallis test was used for continuous variables, and chi-square test was used for categorical variables. LBRs within each complete IVF/ICSI cycle and CLBRs over 1-4 complete cycles were calculated. Log-rank test was used to compare the CLBRs between different age groups and ovarian reserve. COX regression model was further conducted to investigate clinical independent factors associated with CLBRs. Statistical significance was set at *P*<0.05 and the significant level was adjusted using the Bonferroni correction for multiple comparisons. All statistical analyses were performed using the SPSS 22 and SAS 9.3 software.

## Supplementary Material

Supplementary Tables
