# Supplementary material for: Can repeat IVF/ICSI cycles compensate for the natural decline in fertility with age? an estimate of cumulative live birth rates over multiple IVF/ICSI cycles in Chinese advanced-aged population
Source: Aging (Albany NY). 2021 May 20;13(10):14385–98. doi: 10.18632/aging.203055 (PMC8202897; doi:10.18632/aging.203055)
Supplement: Supplementary Tables [file aging-13-203055-s001.pdf]

SUPPLEMENTARY TABLES

Supplementary Table 1. Live-birth rates within each treatment cycle and cumulative live-birth rate over the first five cycles in 3,486 Chinese advanced age women undergoing 5088 cycles of IVF/ICSI.

Cycle number	N cycles	N live-births	Live-birth rate within each cycle % (95%CI)	Cumulative live-birth across first five cycles using different estimates % (95%CI)		
				Optimal estimate[a]	Age-adjusted estimate[b]	Conservative estimate[c]
**1**	3486	1121	32.2 (30.6, 33.7)	32.2 (30.6, 33.7)	32.2 (30.6, 33.7)	32.2 (30.6, 33.7)
**2**	1115	241	21.6 (19.2, 24.0)	46.8 (45.4, 48.3)	43.5 (42.1, 44.9)	39.8 (38.3, 41.2)
**3**	336	43	12.8 (9.2, 16.4)	53.6 (52.2, 55.1)	47.4 (46.0, 48.8)	41.4 (39.9, 42.8)
**4**	113	8	7.1 (2.4, 11.80	56.9 (55.4, 58.4)	48.8 (47.4, 50.2)	41.7 (40.3, 43.1)
**5**	38	1	2.6 (-2.5,7.7)	58.0 (56.5, 59.5)	49.1 (47.8, 50.5)	41.7 (40.3, 43.1)

[a]The optimal estimate assumes that women who discontinued IVF/ICSI treatments would have live-birth rate similar to those continuing treatments.

[b]The age-adjusted estimate assumes that the cumulative live-birth rate in women who discontinued IVF/ICSI, if they had continued, would have been equal to the rate in women who were the same age at the start of treatment, and who continued to have further IVF/ICSI. These results suggested approximately 47% of women who discontinued did so because of poor prognosis and would have had a live-birth rate of zero, had they continued.

[c]The conservative estimate assumes that women who discontinued IVF treatments would have a live-birth rate of zero if they continued treatments.

Supplementary Table 2. Cumulative live birth rates stratified according to ovarian reserve in women aged 35-37yrs.

Cycle number	Retrieval n	Live births	y	live birth rate per cycle (95% CI)	Optimal estimated CLBR (95% CI)	Conservative estimated CLBR (95% CI)
**Non- POSEIDON**						
1	330	144	6	43.6%(38.3,49.0)	43.6%(38.3,49.0)	43.6%(38.3,49.0)
2	86	29	4	33.7%(23.7,43.7)	62.6%(57.0,68.3)	53.0%(48.0,58.1)
3	22	7	1	31.8%(12.4,51.3)	74.5%(66.9,82.1)	55.7%(50.7,60.7)
4	4	0	1	0	74.5%(66.9,82.1)	55.7%(50.7,60.7)
**POSEIDON group 2**						
1	296	63	8	21.3%(16.6,25.9)	21.3%(16.6,25.9)	21.3%(16.6,25.9)
2	140	32	1	22.9%(15.9,29.8)	39.3%(35.4,43.2)	32.7%(28.6,36.8)
3	41	5	1	12.2%(2.2,22.2)	46.7%(42.6,50.8)	34.5%(30.5,38.6)
4	10	2	1	20.0%(-4.8,44.8)	57.3%(52.9,61.8)	35.3%(31.4,39.3)
**POSEIDON group 4**						
1	146	22	4	15.1%(9.3,20.9)	15.1%(9.3,20.9)	15.1%(9.3,20.9)
2	62	10	5	16.1%(7.0,25.3)	28.8%(23.7,33.8)	22.4%(17.0,27.7)
3	25	4	1	16.0%(16.3,30.4)	40.2%(35.3,45.0)	25.9%(20.7,31.0)
4	10	1	0	10.0%(-8.6,28.6)	46.1%(40.9,51.4)	26.8%(21.7,31.9)

Supplementary Table 3. Cumulative live birth rates stratified according to ovarian reserve in women aged 38-39yrs.

Cycle number	Retrieval n	Live births	y	Live birth rate per cycle (95% CI)	Optimal estimated CLBR (95% CI)	Conservative estimated CLBR (95% CI)
**Non-POSEIDON**						
1	984	529	31	53.8%(50.6,56.9)	53.8%(50.6,56.9)	53.8%(50.6,56.9)
2	139	58	3	41.7%(33.5,49.9)	73.1%(68.3,77.8)	61.0%(57.7,64.3)
3	17	5	1	29.4%(7.8,51.1)	81.0%(71.3,90.6)	61.7%(58.4,65.0)
4	2	0	0	0	81.0%(71.3,90.6)	61.7%(58.4,65.0)
**POSEIDON group 2**						
1	721	231	28	32.0%(28.6,35.4)	32.0%(28.6,35.4)	32.0%(28.6,35.4)
2	178	49	7	27.5%(21.0,34.1)	50.7%(47.5,54.0)	40.0%(36.7,43.1)
3	42	10	2	23.8%(10.9,36.7)	62.5%(58.2,66.8)	41.8%(38.7,44.9)
4	5	1	0	20.0%(-15.1,55.1)	70.0%(60.9,79.0)	42.0%(38.9,45.1)
**POSEIDON group 4**						
1	225	32	4	14.2%(9.7,18.8)	14.2%(9.7,18.8)	14.2%(9.7,18.8)
2	95	18	5	18.9%(11.1,26.8)	30.5%(26.6,34.3)	22.6%(18.4,26.7)
3	23	1	2	4.3%(-4.0,12.7)	33.5%(29.4,37.5)	23.1%(19.0,27.3)
4	7	1	0	14.3%(-11.6,40.2)	43.0%(39.4,46.6)	23.8%(19.7,28.0)

Supplementary Table 4. Cumulative live birth rates stratified according to ovarian reserve in women aged 40-42yrs.

Cycle number	Retrieval n	Live births	y	Live birth rate per cycle (95% CI)	Optimal estimated CLBR (95% CI)	Conservative estimated CLBR (95% CI)
**Non-POSEIDON**						
1	159	43	10	27.0%(20.1,33.9)	27.0%(20.1,33.9)	27.0%(20.1,33.9)
2	63	17	3	27.0%(16.0,37.9)	46.7%(40.9,52.6)	39.4%(33.4,45.5)
3	17	2	1	11.8%(-3.6,27.1)	53.0%(46.4,59.6)	41.1%(35.2,47.1)
4	4	2	0	50.0(1.0,99.0)	76.5%(70.0,83.1)	43.3%(37.5,49.1)
**POSEIDON group 2**						
1	228	35	13	15.4%(10.7,20.0)	15.4%(10.7,20.0)	15.4%(10.7,20.0)
2	119	17	9	14.3%(8.0,20.6)	27.4%(23.3,31.6)	23.6%(19.3,27.9)
3	36	6	1	16.7%(4.5,28.8)	39.5%(35.7,43.4)	27.3%(23.2,31.4)
4	14	0	2	0	39.5%(35.7,43.4)	27.3%(23.2,31.4)
**POSEIDON group 4**						
1	207	17	5	8.2%(4.5,12.0)	8.2%(4.5,12.0)	8.2%(4.5,12.0)
2	105	8	5	7.6%(2.5,12.7)	15.2%(11.7,18.7)	12.3%(8.7.15.8)
3	45	1	1	2.2%(-2.1,6.5)	17.1%(13.7,20.5)	12.8%(9.3,16.4)
4	19	1	0	5.3%(-4.8,15.3)	21.5%(18.2,24.7)	13.4%(9.9,17.0)
